# Stem Cell Markers in Neuroblastoma—An Emerging Role for LGR5

**DOI:** 10.3389/fcell.2015.00077

**Published:** 2015-12-02

**Authors:** Helen Forgham, Darren Johnson, Noel Carter, Stephany Veuger, Jane Carr-Wilkinson

**Affiliations:** ^1^Cancer Biology Group, Department of Pharmacy, Health and Well Being, Cancer Biology and Therapeutics, Faculty of Applied Sciences, University of SunderlandSunderland, UK; ^2^Children's Cancer Institute, University of New South WalesSydney, NSW, Australia; ^3^Paediatric Oncology and Haematology, Newcastle Cancer Centre at The Northern Institute for Cancer Research, Newcastle UniversityNewcastle Upon Tyne, UK

**Keywords:** neuroblastoma, LGR5, relapse, chemoresistance

## Abstract

The prognostic value of cancer stem cell markers in various cancer subtypes is a well documented research area. Our findings show that the stem cell marker Lgr5 is associated with an aggressive phenotype in neuroblastoma. Here, we discuss these findings within the context of recent studies in several cancers such as lung, colorectal and intestinal cancer, glioblastoma and ewing's sarcoma. Neuroblastoma continues to be an elusive disease, due to its heterogeneous presentation ranging from spontaneous regression to aggressive metastatic disease and intertwined genetic variability. Currently, the most significant prognostic marker of high risk disease and poor prognosis is amplification of the *MYCN* oncogene, which is found in approximately 25% of cases (Huang and Weiss, 2013). With this in mind, there is still much to learn about the driving mechanisms of this aggressive pediatric tumor. Neuroblastoma development is thought to be the result of aberrant differentiation of the cell of origin, embryonic neural crest cells which then migrate and invade during the developmental stage (Joshi et al., 2007). Aberrant cells are those which would, under normal conditions form the mature tissues of the sympathetic ganglia and adrenal medulla. Tumors are known to develop indiscriminately along the radius of the sympathetic ganglia, although it is well established that the adrenal glands are fundamentally the most common primary site (Jessen and Mirsky, 2005).

## Cancer stem cells

Within recent years there has been an increased focus on cancer stem cells as important drivers in both cancer relapse and in chemoresistance, thereby determining cancer aggressiveness. Elucidation of the mechanisms of how cancers stem cells arise in conjunction with the identification of stem cell markers constitute fundamental steps in understanding oncogenesis, metastasis, and resistance to therapy which are often observed in high risk neuroblastoma (Buhagiar and Ayers, 2015). Cancer stem cells, by definition, possess stem cell like characteristics including enhanced self-renewal, migration, and chemo resistance.

Emerging evidence from several tumor types including neuroblastoma recognizes the potential driving role of such cancer stem cells in disease progression, relapse and poor outcome (Kamijo and Nakagawara, 2012; Khalil et al., 2012; Kuo et al., 2015). The existence of stem like characteristics in tumor cells has been reported in high risk neuroblastomas, a sub-population of cells which exhibit stem cell properties have been isolated “Tumour Initiating Cells” which have the ability to initiate tumor growth (Hansford et al., 2007). Recent data also suggests that cancer stem cells may determine clinical behavior and treatment response in neuroblastoma (Pandian et al., 2015; Ross et al., 2015). Such studies pave the way for the analysis of genes involved in neural crest development to evaluate whether their deregulation in tumors is associated with progression and the emergence of relapsed disease (Wurdak, 2012).

## LGR5 as a stem cell marker

In search of cancer stem cells markers, Barker and Clevers in 2000 uncovered the dogmatic existence of adult stem cell marker Lgr5 also known as G-protein coupled receptor (GPR49) (chromosome 12; position 12q22–q23) (Haegebarth and Clevers, 2009). Structurally, Lgr5 is made up of 907 amino acids; it has a large extracellular domain consisting of 17 leucine-rich repeats and a seven-transmembrane domain (Carmon et al., 2012). Lgr5 transduces extracellular signals through its direct affect with guanine nucleotide-binding (G) protein (Barker and Clevers, 2010). Lgr5 is specifically expressed on cancer stem cells (CSCs) and is known to amplify the effect of Wnt/β-catenin signaling by working as a receptor for R-spondins (RSPO), which are well established and influential agonists of the Wnt pathway (Carmon et al., 2012), and known key drivers of oncogenesis (de Lau et al., 2014).

## The role of LGR5 in cancer the story so far

Lgr5 is a stem cell marker expressed during embryonic development and in adult stem cells in the colon and kidney (Liu et al., 2013). In recent years Lgr5 has been reported to promote both growth and survival and is over expressed in several types of cancer including colorectal cancers (van de Wetering et al., 2002; Barker et al., 2007), where heightened expression was restricted to actively proliferating cells, Ovarian cancer (McClanahan et al., 2006), and Thyroid Cancer (Michelotti et al., 2015). Lgr5 is also of relevance in pediatric tumors including glioblastomas (Nakata et al., 2013) and Ewing's Sarcoma (Scannell et al., 2013). Hermey et al. (1999) also reported the presence of LGR5 in the murine embryonic central nervous system, but not in adult tissue of the same type, which may imply that Lgr5 expression is restricted to neural crest precursors (Hermey et al., 1999).

## Characterization of LGR5 in neuroblastoma

To investigate this potential link between elevated LGR5 expression and aggressiveness in neuroblastoma we performed Q-Real Time PCR to determine LGR5 mRNA expression in a panel of 11 neuroblastoma cell lines displaying a wide range of reported aggressiveness. Expression of LGR5 was quantified using Taqman® gene expression assay primers and probes *(Life Technologies, UK*). Analysis was performed using the ABI Prism 7900 HT sequence detection system *(Applied Biosystems, UK)*, gene expression was quantified relative to GAPDH.

## Elevated expression of LGR5 in a sub-set of cell lines established at relapse and associated with an aggressive phenotype

**Figure 1:**
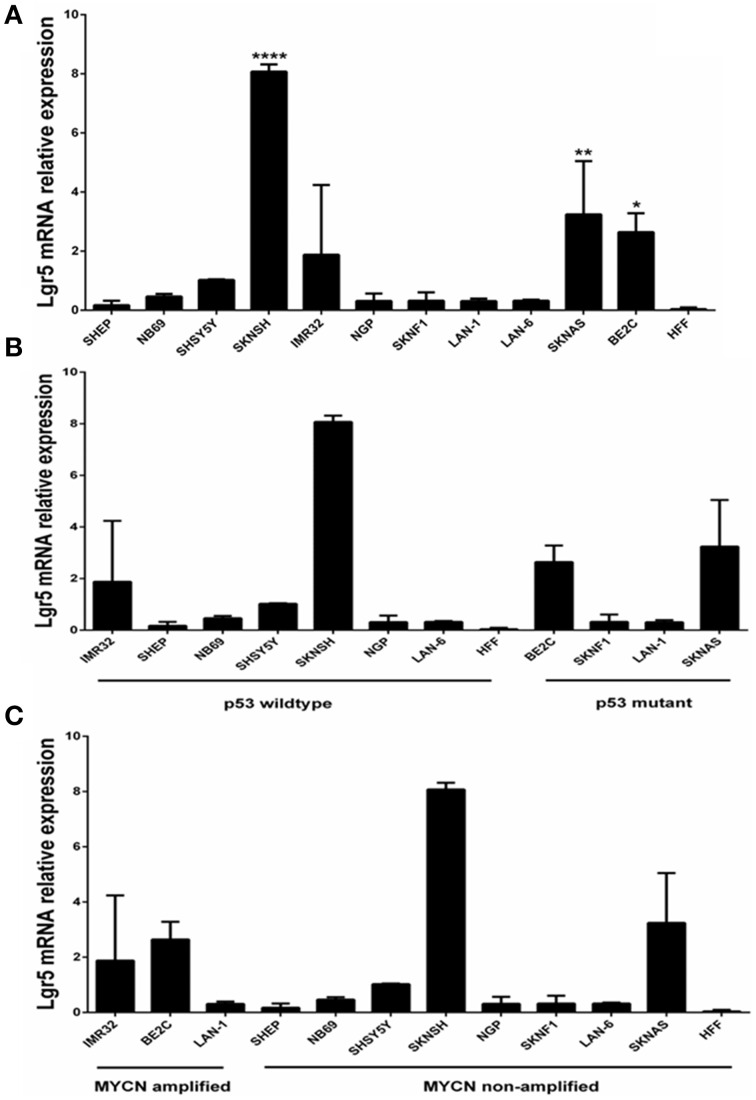
**Quantification of Lgr5 mRNA expression using quantitative RT-PCR. (A)** LGR5 mRNA expression in a panel of 11 neuroblastoma cell lines established at diagnosis or relapse (*N* = 3). Elevated expression of LGR5 was shown in BE2c, SKNAS and ^****^SKNSH (*p* < 0.0001), ^**^SKNAS (*p* < 0.01) and ^*^BE2C (*p* < 0.05) (One-way ANOVA). Human foreskin fibroblasts were used as a control cell line. **(B)** LGR5 mRNA expression in p53 mutant and wild-type neuroblastoma cell lines (*p* = 0.487, Paired *t*-test). **(C)** LGR5 mRNA expression in *MYCN* amplified and non-amplified sub groups. Cell lines were divided into *MYCN* amplified (IMR32, BE2c, and LAN-1) and *MYCN* non-amplified (*p* = 0.339, Paired *t*-test).

Elevated LGR5 mRNA expression was observed in three cell lines BE2c, SKNAS, and SKNSH cell lines (*p* < 0.001) (Figure 1A). High Lgr5 expression was observed in three cell lines (BE2c, SKNSH, and SKNAS), all established from relapsed tumors. Statistical analysis by One-way ANOVA revealed that there was a significant correlation between cell line and expression of Lgr5 *F*_(11, 24)_ = 20.20, *p* < 0.0001. Dunnett's *post-hoc* test revealed a significant difference in the expression of Lgr5 in cell lines SKSNH (*p* < 0.0001), SKNAS (*p* < 0.01), and BE2C (*p* < 0.05) relative to SHEP cells. The SHEP cell line was established from a primary tumor from patient it is a non-aggressive cell line that is *MYCN* non-amplified and p53 wildtype (Carr et al., 2006; Carr-Wilkinson et al., 2011).

To determine whether there was a correlation between Lgr5 and *p53* mutational status cell lines were grouped into mutant and wild type (Figure 1B). The two highest expressers of Lgr5 relative to GAPDH (BE2c and SKNAS) were also proven to be *p53* mutant. Despite differences in the mean expression of LGR5 in p53 mutant and wild type cell lines no statistical significance was observed (*p* = 0.487, Paired *t*-test). To look for a correlation between cells harboring *MYCN* amplification, the cell lines were then categorized by their status. Although differences in the overall mean values were observed, no statistical correlation was observed between *MYCN* amplification status and elevated LGR5 expression (*p* = 0.339, paired *t*-test; Figure 1C).

**Figure 2:**
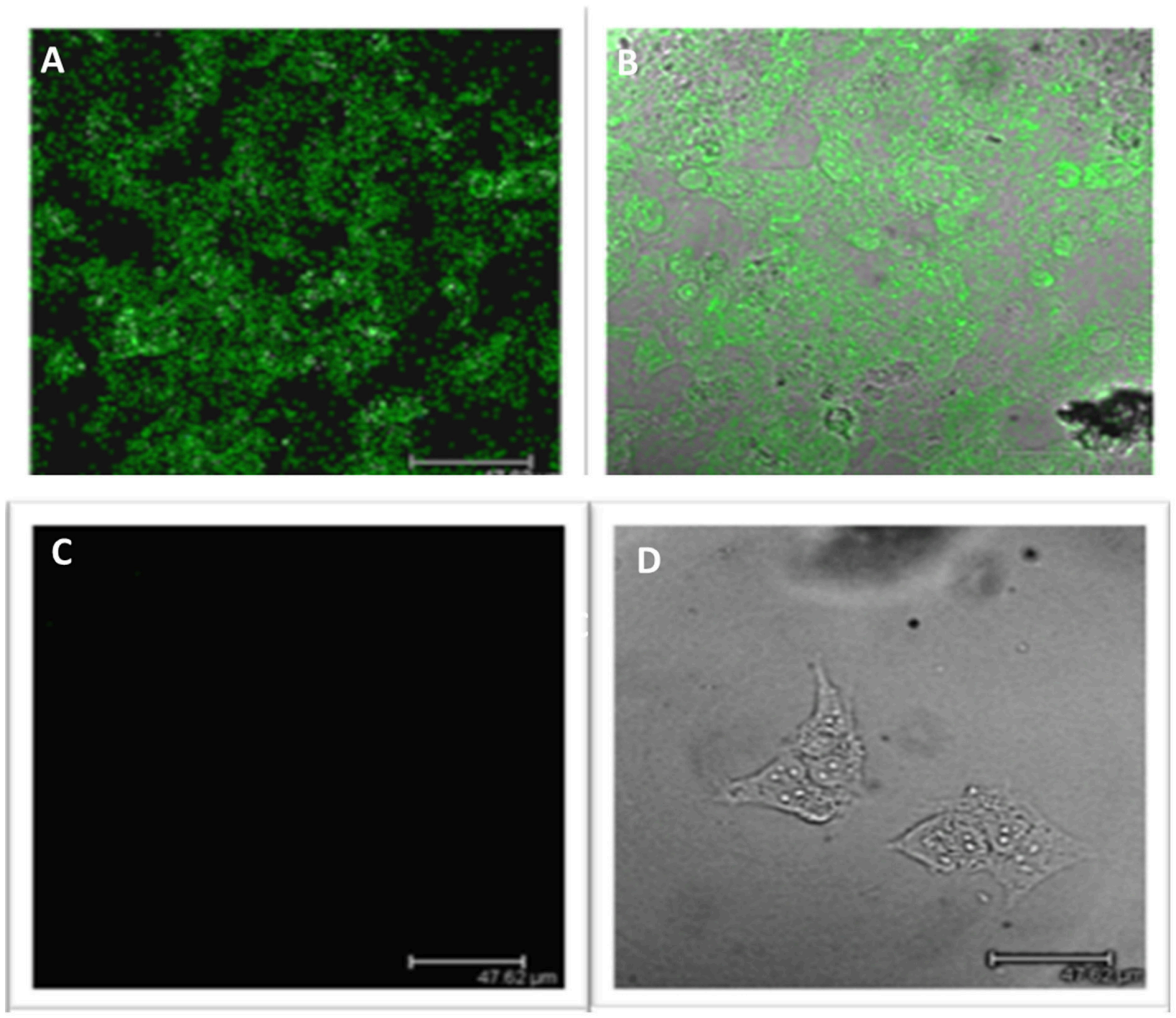
**Immunofluorescence staining of LGR5 in neuroblastoma cell lines. (A)** Immunofluorescence staining of Lgr5 in BE2c cells **(B)** Overlay shows that it is primarily located in the cytoplasm of BE2c neuroblastoma cells **(C)** NB69 cells show an absence of Lgr5 staining when stained under the same conditions as the BE2c cell line, indicating low expression **(D)** Overlay top right shows the morphology of the cell, with the absence of stain by phase contrast microscopy.

The differences in LGR5 expression were also confirmed at a protein level using immunofluorescence on both NB69 and BE2c cell lines (Figure 2). Two out three cell lines with increased LGR5 expression, BE2C and SKNAS, are known to harbor a *p53* mutation, (Carr et al., 2006; Ikegaki et al., 2014) and have been reported to show aggressive chemoresistant behavior (Keshelava et al., 2001; Tweddle et al., 2001).

## LGR5 expression and aggressiveness in neuroblastoma

To further investigate the potential role of LGR5 in neuroblastoma publically accessible data bases were probed. Recent microarray data deposited in the R2 Neuroblastoma tumor database (http://r2.amc.nl) and (NCBI- Geo) show high expression of LGR5 in *MYCN* amplified tumors compared with non-amplified tumors (ANOVA *p* < 0.0001), high expression of LGR5 was also associated with disease recurrence/progression (ANOVA *p* = 0.0013) as well as stage 4 and 4s disease (ANOVA *p* = 0.02). Stage 4 disease is characterized by metastatic aggressive disease types whereas Stage 4s although metastatic at diagnosis affects infant >18 months (http://r2.amc.nl). Microarray data from the mutated ALK driven neuroblastoma mouse model showed an increase in LGR5 expression in aggressive *MYCN* amplified tumors as well as aggressive tumors with both *MYCN* amplification and mutated ALK which is in agreement with the data presented in our study that LGR5 is highly expressed in human cell lines derived from aggressive relapsed cell lines (http://www.ncbi.nlm.nih.gov/geoprofiles/101299025). This data further support the hypothesis that LGR5 may be a potential biological driver of disease progression and relapse in neuroblastoma.

## LGR5 expression as a marker of aggressive disease

Our study shows high LGR5 expression in SKNSH cells, which have been reported to be highly chemoresistant (Blaheta et al., 2007). Given that SKNSH are reported to spontaneously switch between S and N type cells (Ganeshan and Schor, 2014), further investigation into how Lgr5 may play a potential role in this spontaneous transition may lead to new insights into tumor survival and proliferation.

Our findings are important and in agreement with Scannell et al. (2013) who showed that Lgr5 levels were significantly raised in cell lines derived from metastatic disease as well as in biopsies taken from initial diagnosis, where patients quickly developed untreatable disease. Their study showed that undifferentiated neural crest stem cell expression of Lgr5 was substantially higher than expression in cells differentiating into mesenchymal stem cells (Scannell et al., 2013). A more recent study in glioblastoma identified LGR5 as a marker of poor prognosis and a molecule playing an instrumental role in the survival of brain cancer stem-like cells; stem cell marker CD133+ sorted cells expressed higher levels of LGR5 than the CD133 negative cell populations and upon differentiation, LGR5 expression was significantly repressed (Nakata et al., 2013). Upon further investigation it was also noted that inhibiting expression at a genetic level resulted in suppression of proliferation and cell death (Wang et al., 2014).

It is conceivable that there is a link between Lgr5 expression and maturation status of neural crest embryonic stem cells and that high LGR5 expression is associated with more aggressive phenotypes and increased relapse rate. Interestingly, in neuroblastoma, Balamuth et al. (2010) identified increased expression of Lgr5 (Gpr49) as a potential therapeutic target and marker of disease in the murine TH-MYCN neuroblastoma model and in human *MYCN* amplified neuroblastomas (Balamuth et al., 2010).

Taken together these studies indicate a potential role of LGR5 in neuroblastoma cancer stem cell development, warranting further investigation into this protein in this disease.

Our findings support a growing body of evidence associating LRG5 with aggressiveness in several cancer subtypes. Our investigation shows high expression of LGR5 in the *MYCN* amplified BE2C cell line, which previous studies reported to be the most aggressive cell type in the panel of cells under investigation (Keshelava et al., 2001; Tweddle et al., 2001). Interestingly, the BE2C cell line is reported to comprise exclusively of I type cells (Walton et al., 2004). Walton et al. (2004) reported that cellular phenotype was an important determinant of malignant potential in neuroblastoma and identified a stem cell phenotype in I-type cells that was shown to be 4–5 times more tumorigenic than N- and S-type cells. In the same study immunocytochemistry of tumor sections from patients with progressive disease exhibited higher numbers of I-like cells than those biopsied from patients with progression- free survival (Walton et al., 2004). More recently high expression of stem cell related genes was shown in highly malignant I-type neuroblastoma cells which could potentially imply that these cells comprise specific sets of proteins which enable sustained proliferation and enhanced survival (Ross et al., 2015).

## Conclusion and future directions

In summary, we have shown elevated expression of LGR5 in a subset of neuroblastoma cell lines which are derived from patients at relapse and display an aggressive phenotype. These data, in combination with available microarray data study, which strongly support our findings, suggest a pressing need for further investigation of the role of LGR5 and other stem cell markers in tumor progression and as a prognostic biomarker in pediatric tumors such as neuroblastoma, as well as other cancer sub-types. Furthermore, these data imply that stem cell markers including LGR5 may be a potential biomarker for aggressive disease or important drivers of disease relapse and drug resistance. Importantly, we demonstrate that this is an important area of investigation and it is clear that a better understanding of the role of stem cell related genes is required. Further understanding would herald the potential for novel therapeutic intervention to increase patient survival for childhood tumors, particularly aggressive high risk neuroblastoma.

## Funding

University of Sunderland Research Beacon Grant.

### Conflict of interest statement

The authors declare that the research was conducted in the absence of any commercial or financial relationships that could be construed as a potential conflict of interest.
